# MacroH2A1 knockdown effects on the *Peg3 *imprinted domain

**DOI:** 10.1186/1471-2164-8-479

**Published:** 2007-12-31

**Authors:** Jung Ha Choo, Jeong Do Kim, Joomyeong Kim

**Affiliations:** 1 Department of Biological Sciences, Louisiana State University, Baton Rouge, LA, 70803, USA; 2 Department of Biological Sciences, Korea Advanced Institute of Science and Technology, Daejeon, 305-701, South Korea

## Abstract

**Background:**

MacroH2A1 is a histone variant that is closely associated with the repressed regions of chromosomes. A recent study revealed that this histone variant is highly enriched in the inactive alleles of Imprinting Control Regions (ICRs).

**Results:**

The current study investigates the potential roles of macroH2A1 in genomic imprinting by lowering the cellular levels of the macroH2A1 protein. RNAi-based macroH2A1 knockdown experiments in Neuro2A cells changed the expression levels of a subset of genes, including *Peg3 *and *Usp29 *of the *Peg3 *domain. The expression of these genes was down-regulated, rather than up-regulated, in response to reduced protein levels of the potential repressor macroH2A1. This down-regulation was not accompanied with changes in the DNA methylation status of the *Peg3 *domain.

**Conclusion:**

MacroH2A1 may not function as a transcriptional repressor for this domain, but that macroH2A1 may participate in the heterochromatin formation with functions yet to be discovered.

## Background

The nucleosome, the basic unit of eukaryotic chromatin, consists of a 146-bp DNA wrapped around a histone octamer composed of two copies of core canonical histones, H2A, H2B, H3, and H4. Eukaryotic chromatin also contains a small amount of atypical histones, which have different amino acid sequences than canonical histones, including CENPA, H3.3, H2A.X, H2A.Z, and macroH2A [1]. Among these histone variants, the macroH2A variants display the most unusual protein structure: an N-terminal H2A domain is fused to a large C-terminal non-histone domain. This results in a histone that is nearly three times the size of conventional histones [2]. The N-terminal third of macroH2A (H2A-like) shares 64% sequence identity with H2A, and the remaining two thirds of the protein shows similarity with the domain called 'macro' [3]. MacroH2A is conserved throughout all vertebrate lineages, and two different members of the macroH2A family have been identified to date, macroH2A1 and macroH2A2 [2,4]. The macro domain functions as a strong transcriptional repressor by inhibiting the initiation step of Pol II transcription and by interfering with p300-dependent histone acetylation [5]. The macro domain is also capable of blocking the chromatin remodeling process mediated by SWI/SNF and ACF [5,6].

MacroH2A is mainly associated with the heterochromatic regions of chromosomes. This is consistent with the observed repression activity. Notably, early immuno-fluorescence studies revealed the enrichment of macroH2A1 deposition in the inactive X chromosome of mammals [7,8], which undergoes chromosome-wide repression to balance different gene dosage between female and male [9]. MacroH2A1 is also associated with other regions of heterochromatin, including peri-centromeric regions and senescence-associated heterochromatic foci [10-12]. Another recent study revealed that high levels of macroH2A1 deposition are detected in methylated CpG islands that are located close to promoter regions [13]. In particular, macroH2A1 is highly enriched in the inactive, methylated alleles of Imprinting Control Regions (ICRs), which are shown to be critical for maintaining the imprinting (allele-specific expression) of surrounding genes [14,15]. Although the mechanism(s) targeting macroH2A1 to the ICRs are not well understood at moment, the allele-specific deposition of macroH2A1 in the ICRs represents another epigenetic marker besides DNA methylation that differentiates two parental alleles.

To characterize potential roles of macroH2A1 in genomic imprinting, the current study has analyzed several imprinted domains with a major focus on the *Peg3 *domain, an evolutionarily well-conserved domain located in human chromosome 19q13.4/proximal mouse chromosome 7 [16]. This domain contains 6 imprinted genes within a 500-kb genomic interval, including paternally expressed *Peg3*, *Usp29 *and *Zfp264*, and maternally expressed *Zim1*, *Zim2 *and *Zim3*. We have generated several stable cell lines showing low levels of macroH2A1 using Neuro2A cells and analyzed changes in the expression levels of imprinted genes. The results indicate that a subset of imprinted genes, including *Peg3 *and *Usp29*, were affected by lowering the cellular levels of the macroH2A1 protein. Furthermore, the expression of these genes were further repressed, rather than de-repressed, in response to the knockdown of the potential repressor macroH2A1. This suggests that macroH2A1 may not function as the dominant repressor for the transcription of imprinted genes.

## Results

### MacroH2A1 knockdown stable cell lines

**Figure 1:**
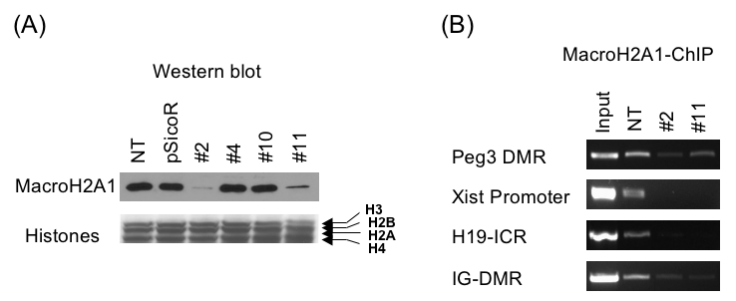
**MacroH2A1 knockdown cell lines by siRNA**. **(A) **MacroH2A1 protein levels in knockdown cell lines. Histone extracts were prepared from six different types of Neuro2A cells: those were not transfected (NT), transfected with the empty pSicoR vector (pSicoR), or transfected with macroH2A1 siRNA constructs (#2, #4, #10 and #11). These histone extracts (5 μg per each well) were separated on 14% SDS-PAGE and analyzed with western blotting (Top panel). Even loading and transfer of the histone extracts was monitored by staining with Coomassie Blue (Bottom panel). **(B) **Chromatin immunoprecipitation analysis of macroH2A1 in the differentially methylated regions (DMRs) and promoters of several imprinted genes. IG-DMR (Intergenic DMR) is an ICR located in the Dlk1/Gtl2 imprinted domain whereas H19-ICR is an ICR located 2-kb upstream of H19. Individual ChIP analyses were performed using the three types of cells: without transfection (NT) and the two stable transfectants (#2 and #11). The amplified PCR products derived from immunoprecipitated DNAs were compared with those amplified from the chromatin DNA before immunoprecipitation (Input, 10%). Since three individual ChIP experiments derived similar amounts of Input DNAs, a representative Input from an NT sample is shown.

To investigate potential roles of macroH2A1 in genomic imprinting, we decided to lower the cellular levels of macroH2A1 using siRNA techniques and to analyze functional outcome of this knockdown. We designed four siRNA constructs using the pSicoR vector system [17], and tested the efficacy of these constructs by performing individual transient transfection and western blotting (data not shown). Despite several trials, however, this series of transient experiments were not successful mainly due to the relatively high levels and long half-life of the cellular macroH2A1 protein. Thus, we decided to derive stable cell lines using these constructs. We transfected a pool of these four constructs into both NIH3T3 and Neuro2A cells along with pcDNA3.1/His/lacZ vector (Invitrogen) for G418 selection (Fig. 1A). We initially seeded about 200 single cells per each line, and successfully obtained 20 homogenous pools of single cell lines that still maintained the EGFP expression (an indicator for the siRNA expression) from both NIH3T3 and Neuro2A. However, we decided to analyze only the stable cell lines derived from Neuro2A due to the following two reasons. First, Neuro2A is a cell line with female and neuron cell origins, which is appropriate for our expression studies on imprinted genes with neuron specificity as well as *Xist *with female specificity. Second, we have been able to extract histones more consistently from Neuro2A than NIH3T3, which is a crucial step for measuring accurate knockdown levels of macroH2A1. Out of 4 randomly chosen single cell lines, two cell lines (#2 and #11) consistently showed the reduced levels of macroH2A1, approximately 80 to 90% reduction. In contrast, the remaining two cell lines (#4 and #10) did not show any change in the protein levels of macroH2A1. Therefore, two cell lines, #2 and #11, have been selected for our following analyses.

We performed chromatin immunoprecipitation (ChIP) experiments using the two macroH2A1 knockdown cell lines to test whether macroH2A1 is indeed depleted in the Imprinting Control Regions (ICRs) of several domains, which show high levels of macroH2A1 deposition [13]. According to several ChIP experiments using anti-macroH2A1 antibody, the deposition levels of macroH2A1 on ICRs in the two stable cell lines were much lower than the levels detected in the positive control Neuro2A cells (Fig. 1B). This confirms the depletion of macroH2A1 in most ICRs in the two macroH2A1-knockdown cell lines. The growth and morphology of these stable cell lines appeared to be normal without any obvious abnormalities.

### MacroH2A1 knockdown effects on the transcription of imprinted genes

**Figure 2:**
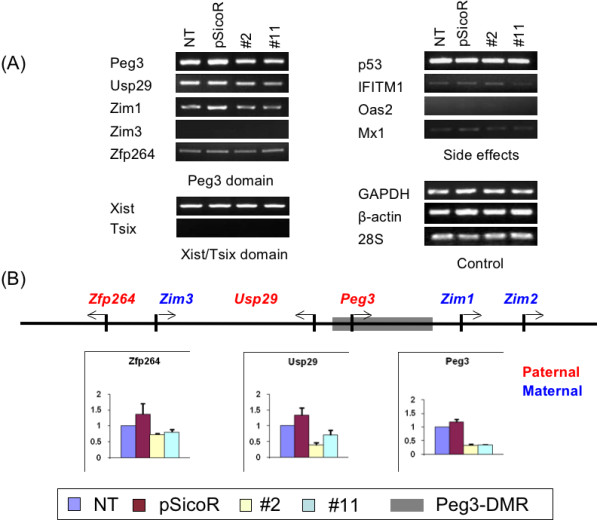
**Expression level changes of imprinted genes in macroH2A1 knockdown cell lines**. **A) **The expression levels were measured by RT-PCR with fixed number of cycles ranging from 30 to 38. The cycle numbers for *Zim1 *and *Tsix *were 38 and 36, respectively, and the cycle numbers for all the remaining genes were 34. The amplified products from four different cell lines were separated on agarose gels: lane 1, the Neuro2A cells with no transfection (NT); lane 2, the transfectants with the empty pSicoR vector (pSicoR); lane 3, the stable transfectant #2; and lane 4, the stable transfectant #11. Individual genes are grouped together based on their chromosomal locations (*Peg3 *and *Xist/Tsix *domains) and their purposes (Side Effects and Control). *GAPDH*, *β-actin *and *28S *were used as quantitative controls. Four genes, including *p53*, *IFITM1*, *Oas2 *and *Mx1*, were used for monitoring potential toxic and side effects caused by siRNA experiments. **B) **Quantitative real time PCR analysis of the genes located within the *Peg3 *domain. The genomic structure of the *Peg3 *domain illustrated with gene names, lines and arrows. Gene names in red color represent paternally expressed genes while those in blue color represent maternally expressed genes. The expression levels of each gene were first normalized with two different control genes, *GAPDH *and *28S*, and later compared with the normalized level of the cells without transfection (NT). The values in graphs are the averaged fold differences relative to those of the non-transfected cells (NT) with standard deviations (S.D.). We performed this experiment at least three times from RNA isolation to real time qRT-PCR.

The potential effects of macroH2A1 knockdown on imprinted domains were investigated by analyzing the expression levels of several genes in the two macroH2A1 knockdown cell lines. For this series of experiments, we first isolated total RNAs from 4 different cell lines: Neuro2A with no transfection (NT), transfection with the empty pSicoR vector (pSicoR), stable cell line #2 and #11. The isolated RNAs were reverse transcribed and analyzed with two independent experiments: RT-PCR with a fixed number of cycles, ranging from 30 to 38 (Fig. 2A), and quantitative real time PCR (Fig. 2B). To normalize cDNA amounts, three genes were selected as internal controls (*GAPDH*, *28S*, and *β-actin*). We also monitored potential toxic and side effects of siRNA experiments, such as interferon response, by checking the expression levels of four genes (*p53*, *IFITM1*, *Oas2 *and *Mx1*) (Fig. 2A).

In the *Peg3 *domain, the expression of most resident genes except for *Zim3 *was detected in the Neuro2A-derived control and knockdown cell lines. The expression levels of *Peg3 *and *Usp29 *in the two macroH2A1 knockdown cell lines were lower than those in the two control cell lines (Fig. 2A). The results from qRT-PCR also derived a similar conclusion: the expression levels of *Peg3 *and *Usp29 *were decreased by 2.8- to 3.0-fold and 1.4- to 2.5-fold, respectively (Fig. 2B). This is an unexpected outcome given the prediction that macroH2A1 is a transcriptional repressor [5,6]. However, the expression levels of *Zim1 *and *Zfp264 *did not deviate significantly from those of the control cells (Student's *t *test, 0.05 <*p *< 0.1), indicating that these two genes are probably not changed by macroH2A1 knockdown.

We also analyzed the expression levels of other imprinted genes, including *Gtl2*/*Dlk1*, *Igf2*/*H19, Kcnq1/Kcnq1ot1, Nesp/Nespas, GnasXL, Exon1A*, and *Xist*/*Tsix *(Fig. 2A and see Additional file 1). These genes were analyzed with RT-PCR with a fixed number of cycles, ranging from 30 to 38, after the normalization of cDNAs with the three internal controls (*GAPDH*, *28S*, and *β-actin*). The expression levels of *Gtl2, H19, Kcnq1, Kcnq1ot1*, and *Tsix *were relatively low in the Neuro2A-derived cell lines, and thus we could not analyze the knockdown effects on these genes. The expression levels of *Igf2*, *Nesp *and *GnasXL *in the two knockdown cells were lower than those in the two control cells, indicating potential knockdown effects on these genes. However, due to inconsistent expression levels of these genes in one of the control cells (pSicoR in Fig. 2 and see Additional file 1), we could not confirm the knockdown effects on these genes with statistical significance. In contrast, *Xist *was highly expressed in Neuro2A-derived cell lines, but showed a similar level of expression among all the cell lines analyzed (Fig. 2A). This indicates that macroH2A1 knockdown had no obvious effect on this locus. The expression levels of DNA methyltransferases (Dnmts) were also analyzed in the macroH2A1 knockdown cells. *Dnmt1*, *Dnmt3a *and *Dnmt3b *were expressed at a similar level in both controls and macroH2A1 knockdown cells, showing that macroH2A1 knockdown had no effect (see Additional file 1). In conclusion, our analyses indicate that the expression levels of two genes located in the *Peg3 *domain were down-regulated in the macroH2A1 knockdown cells.

### MacroH2A1 knockdown effects on the DNA methylation of imprinted genes

**Figure 3:**
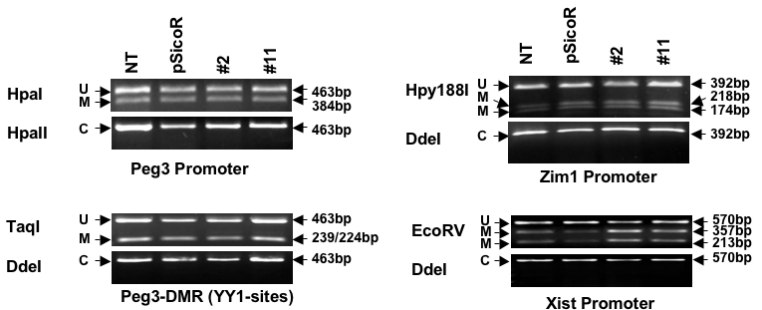
**DNA methylation levels of imprinted genes in macroH2A1 knockdown cell lines**. Genomic DNAs isolated from 4 different cell lines were first treated with sodium bisulfite and later used for PCR amplification. Each PCR product was digested with a set of two enzymes, which are listed on the left column. The enzyme shown on the bottom panel is for monitoring the efficiency of the bisulfite conversion reaction. The un-digestion by these enzymes indicates the complete conversion reaction (C) by the bisulfite reaction. The enzyme shown on the top panel is for measuring the methylation level of each amplified product: the un-digestion by these enzymes indicates un-methylation (U), whereas the digestion indicates methylation (M). The sizes of original PCR products and their digested products are indicated in the right column with arrows.

Since two genes in the *Peg3 *domain were repressed in the knockdown cell lines, we investigated if DNA methylation was involved in this repression. We analyzed the DNA methylation status of several promoters of genes in the *Peg3 *domain using the combined bisulfite restriction analysis (COBRA) [18] (Fig. 3). Genomic DNAs from the control and macroH2A1 knockdown cell lines were isolated, treated with bisulfite, and finally amplified with specific primer sets designed for bisulfite-converted DNAs. To monitor the efficiency of the conversion reaction, each PCR product was first digested with one of the following restriction enzymes, *Hpa*II, and *Dde*I, the recognition sites of which contain a cytosine base (the bottom panel for each product in Fig. 3). Digestion of an amplified product by either one of these enzymes indicates inefficient conversion reaction in a given trial. As shown in Fig. 3, however, none of products were digested, confirming successful conversion by our bisulfite treatment.

To determine the methylation status of promoter regions, we subsequently digested each PCR product with a second series of enzymes that can differentiate methylated and unmethylated DNAs, including *Hpa*I, *Taq*I, *Hpy*188I, and *Eco*RV (the top panel in Fig. 3). Digestion of each amplified product with one of these enzymes indicates DNA methylation, whereas non-digestion indicates that the tested DNA is unmethylated. As shown in Fig. 3, all the tested promoter regions within the *Peg3 *domain derived an equal ratio of methylated to unmethylated DNAs in all the cell lines tested. This is an expected outcome given that only one of two parental alleles of these regions is usually methylated *in vivo *(thus named DMRs, Differentially Methylated Regions). However, we did not find any difference between the DNA methylation levels of the control and knockdown cell lines, suggesting that DNA methylation status of the promoters located in the *Peg3 *domain was not changed in the macroH2A1 knockdown cell lines. This is also true for the *Xist *promoter (Fig. 3). Overall, our DNA methylation analyses indicate that macroH2A1 depletion did not cause any change in the DNA methylation status of imprinted domains.

## Discussion

The current study demonstrates that lowering the macroH2A1 protein levels affects the expression levels of a subset of imprinted genes, including *Peg3 *and *Usp29*. Interestingly, the expression of these genes was repressed, rather than de-repressed, in response to the knockdown of the potential repressor macroH2A1. Subsequent analyses also revealed that macroH2A1 knockdown did not change the DNA methylation status of their promoter regions. This suggests that macroH2A1 may not function as a dominant repressor for the transcription of these imprinted genes.

The down-regulation of a subset of imprinted genes, *Peg3 *and *Usp29*, in macroH2A1 knockdown cells is an unexpected outcome since several lines of evidence strongly suggest a repressor role for this histone variant [5,6]. According to the results from a previous study [13], macroH2A1 is deposited mainly on the inactive allele of the promoter region of *Peg3 *and *Usp29*, suggesting a potential role in the inactive allele of this imprinted domain. RNAi-based knockdown indeed depleted macroH2A1 from the inactive allele of *Peg3 *and *Usp29 *as shown in Fig. 1B. Despite this depletion, however, the inactive allele of the two genes was still methylated at similar levels between the control and knockdown cells (Fig 3). This suggests that macroH2A1 knockdown does not have any impact on the methylation levels and subsequently silent state of the inactive allele. Nevertheless, the expression levels of *Peg3 *and *Usp29 *were down-regulated in the macroH2A1-knockdown cells (Fig. 2). Since macroH2A1 is mainly associated with the inactive allele, the transcription status of which is unaffected in the knockdown cells, the observed down-regulation is likely caused by some changes that have occurred in the active allele. It is, therefore, most likely that the observed effect on *Peg3 *and *Usp29 *in the knockdown cells is an indirect outcome involving other unknown components at their active alleles.

Despite the high levels of macroH2A1 deposition in the ICRs of most known imprinted domains [13], only two imprinted genes, *Peg3 *and *Usp29*, are affected in the macroH2A1-knockdown cell lines. This unexpected outcome is also similar to the results from the macroH2A1-deficient mouse [19]. Depletion of macroH2A1 appears to have affected the transcription of only a small number of genes, about 100 genes, although macroH2A1 has been predicted to be a global regulator. Out of 100 genes, some were also down-regulated, indicating another indirect effect similar to that seen in the macroH2A1-knockdown cells. Since YY1 is known to be involved in the transcription of the two imprinted genes, *Peg3 *and *Usp29 *[20,21], we first analyzed the macroH2A1-knockdown effects on the expression levels of YY1 to obtain potential clues regarding the down-regulation of *Peg3 *and *Usp29*. However, we did not observe any change in the expression level of this potential candidate (see Additional file 1). We also examined the list of genes that were affected in the macroH2A1-deficient mouse [19]. Among the 100 genes affected by macroH2A1 knockout, the transcription factor USF2 (Upstream Stimulating Factor 2) is one likely candidate that was shown to be down-regulated in the macroH2A1-knockout mouse. This transcription factor deserves to be studied further in the near future, since it is known to interact or compete with YY1 for binding to DNA elements [22]. It is plausible that macroH2A1 knockdown might have down-regulated the transcription of *Peg3 *and *Usp29 *through other unknown transcription factors like USF2. Overall, the results described above suggest that macroH2A1 may be involved in the fine tuning of transcriptional control of a subset of genes, but not as a global regulator.

Mammalian imprinted domains are usually co-regulated through a small number of controlling regions, called ICRs [14,15]. Similarly, within the *Peg3 *domain, the surrounding regions of Peg3's 1^st ^exon, termed the Peg3-DMR, are predicted to control the whole domain (Fig. 2B). This potential ICR is characterized by high levels of macroH2A1 deposition as well as an unusual tandem array of YY1 transcription factor binding sites [20,21]. Interestingly, independent knockdown experiments targeting macroH2A1 and YY1 both appear to have resulted in similar global changes in the transcriptional levels of most imprinted genes in this domain. The two imprinted genes of the *Peg3 *domain displayed a down-regulation in response to the macroH2A1 knockdown, although this down-regulation appears to be an indirect outcome. Also, lowering the cellular level of the YY1 protein has resulted in up-regulation of most of the imprinted genes within the *Peg3 *domain [23]. Although opposite, these similar global responses are consistent with the prediction that the imprinting and transcription of this domain is linked together and regulated by a shared mechanism, possibly through the Peg3-DMR.

## Conclusion

RNAi-based macroH2A1 knockdown experiments in Neuro2A cells changed the expression levels of a subset of genes, including *Peg3 *and *Usp29 *of the *Peg3 *domain. The expression of these genes was down-regulated, but not up-regulated, in response to reduced protein levels of the potential repressor macroH2A1. This down-regulation was not accompanied with changes in the DNA methylation status of the *Peg3 *domain. Thus, it is likely that macroH2A1 may not function as a transcriptional repressor for this domain, but that macroH2A1 may participate in the heterochromatin formation with functions yet to be discovered.

## Methods

### MacroH2A1 knockdown using siRNA technique

We designed four siRNA constructs for macroH2A1 knockdown. The sequences of these siRNA constructs are as follows: siRNA1, sense strand, 5'-TGTGCTCGCTTCGGCAGCACATATACTGGCCACCCTAAGTATAGGATTCAAGAGATCCTATACTTAGGGTGGCCTTTTTTC-3'; siRNA1, antisense strand, 5'-TCGAGAAAAAAGGCCACCCTAAGTATAGGATCTCTTGAATCCTATACTTAGGGTGGCCAGTATATGTGCTGCCGAAGCGAGCACA-3'; siRNA2, sense strand, 5'-TGTGCTCGCTTCGGCAGCACATATACTGAGACAACAAGAAGGGACGTTCAAGAGACGTCCCTTCTTGTTGTCTCTTTTTTC-3'; siRNA2, antisense strand, 5'-TCGAGAAAAAAGAGACAACAAGAAGGGACGGTCTCTTGAACGTCCCTTCTTGTTGTCTCAGTATATGTGCTGCCGAAGCGAGCACA-3'; siRNA3, sense strand, 5'-TGTGCTCGCTTCGGCAGCACATATACTACTGCTTGGCTCTAGCTGATTCAAGAGATCAGCTAGAGCCAAGCAGTTTTTTTC-3'; siRNA3, antisense strand, 5'-TCGAGAAAAAAACTGCTTGGCTCTAGCTGATCTCTTGAATCAGCTAGAGCCAAGCAGTAGTATATGTGCTGCCGAAGCGAGCACA-3'; siRNA4, sense strand, 5'-TGTGCTCGCTTCGGCAGCACATATACTTGATGAAGAGCTAAACCAGTTCAAGAGACTGGTTTAGCTCTTCATCATTTTTTC-3'; siRNA4, antisense strand, 5'-TCGAGAAAAAATGATGAAGAGCTAAACCAGTCTCTTGAACTGGTTTAGCTCTTCATCAAGTATATGTGCTGCCGAAGCGAGCACA-3'. Double stranded oligonucleotides were inserted into the *Hap*I and *Xho*I sites of the pSicoR vector [17]. We purified these construct DNAs from *E. coli *using the Hispeed plasmid midi kit (Qiagen), and transfected using the GeneJuice transfection reagent (Novagen) according to the manufacturer's protocol. To derive macroH2A1 knockdown cell line, we co-transfected pcDNA 3.1/His/LacZ vector (Invitrogen) containing the neomycin resistant gene. Transfectants were selected by adding G418 (500 μg/ml; Calbiochem) to the culture medium. After transfection of Neuro2A cells with a pool of the four siRNA constructs, we performed PCR-based cloning and DNA sequencing analyses with DNAs derived from each stable cell line to determine which construct(s) was the most potent in terms of macroH2A1 knockdown. Our analyses indicated that the siRNA #3 and #4 constructs were much more potent than the siRNA #1 and #2 constructs.

### Histone extraction and Western blot

Histones were extracted with 0.2N HCl from 2 × 10^6 ^cells after cell lysis with the triton exaction buffer (0.5% Triton X-100, 2 mM PMSF, 0.02% NaN_3 _in PBS). Protein concentrations were determined by Bradford assay kit (Pierce). Each sample of histone extracts (20 μg) was separated on 14% SDS-PAGE gels, transferred to the PVDF membrane (Hybond-P; Amersham) using a Mini Trans-Blot transfer Cell (Bio-Rad). Membranes were blocked for 1 hour in Tris-buffered saline (TBS-T) containing 5% skim milk and 0.05% Tween-20, incubated at 4°C overnight with anti-macroH2A1 antibody (Cat. # 07-219; Upstate Biotech.). These membranes were further incubated for 1 additional hour with the secondary antibody linked to horseradish peroxidase (Sigma). The blots were visualized with a Western blot detection system (Intron Biotech) according to the manufacturer's protocol.

### RT-PCR and quantitative real time PCR

Total RNAs were isolated from transfectants using Trizol (Invitrogen) and were reverse transcribed using the SuperScript First-Strand Synthesis System (Invitrogen). PCR amplifications were performed with a series of specific primer sets using the Maxime PCR premix kit (Intron Biotech). Quantitative real time PCR was also performed with the iQ SYBR green supermix (Bio-Rad) using the icycler iQ™ multicolor real-time detection system (Bio-Rad). All qRT-PCR were carried out for 40 cycles under the standard PCR conditions. The results derived from qRT-PCR were analyzed based on threshold cycle (Ct) values. Briefly, a ΔCt was first calculated by subtracting the averaged Ct value of two internal controls (*GAPDH *and *28S*) from the averaged Ct value of each gene, and later a ΔΔCt value was calculated by subtracting the two ΔCt values of the targeted gene derived from the Neuro2A-derived knockdown cell and control cell lines. Fold differences were determined by raising 2 to the ΔΔCt power [24]. The information regarding individual primer sequences and PCR conditions are available upon request.

### COBRA (Combined bisulfite restriction analysis)

Genomic DNAs were purified from cell lines using DNAzol (Invitrogen), and 2 μg of each genomic DNA was treated for the bisulfite conversion reaction according to the manufacturer's protocol (EZ DNA methylation kit, Zymo Research). The resultant single-stranded DNAs were used as templates for the PCR reaction using specific primers that were designed for the C-to-T converted DNAs. PCR reaction was performed with the Maxime PCR premix kit (Intron Biotech). Each PCR product was digested with a series of restriction enzymes, first to monitor the efficiency of the bisulfite conversion reaction and later to analyze the methylation status of each region. The restriction enzymes for each region are shown in Fig. 3. The oligonucleotide sequences used for this study are available upon request.

### Chromatin immunoprecipitation (ChIP) analysis

Chromatin immunoprecipitations were performed according to the protocol provided by Upstate Biotechnology (Upstate Biotech) with slight modification. Briefly, we used 2 × 10^7 ^cells from Neuro2A and two macroH2A1 knockdown stable cell lines [2 and #11]. Formaldehyde was added to the culture medium to a final concentration of 1%, and incubated at 37°C for 10 mins. Treated samples were sheared by sonication and immunoprecipitated with anti-macroH2A1 polyclonal antibody (Cat. # 05-457; Upstate Biotech.). Precipitated chromatins were reverse cross-linked and the DNAs were purified by the phenol/chloroform extraction method. Purified DNAs were used as templates for PCR amplification. PCR reactions were carried out for 35 cycles using standard PCR conditions. The resulting PCR products were analyzed by separating on a 1.5% agarose gel. The oligonucleotide sequences used for this study are available upon request.

## Authors' contributions

JHC performed the experiments and wrote the paper, JDK contributed to establish macroH2A1 knockdown cell lines and protein works, and JK provided the original concept of the study, supervised the study and contributed to writing the paper. All authors read and approved the final manuscript.

## Supplementary Material

Additional file 1 MacroH2A1 knockdown effects on the transcription of additional genes. The data represent the macroH2A1-knockdown effects on the expression levels of the genes analyzed in this study. Click here for file
