# Supplementary figures and images for: MacroH2A1 knockdown effects on the Peg3 imprinted domain
Source: BMC Genomics. 2007 Dec 31;8:479. doi: 10.1186/1471-2164-8-479 (PMC2241636; doi:10.1186/1471-2164-8-479)

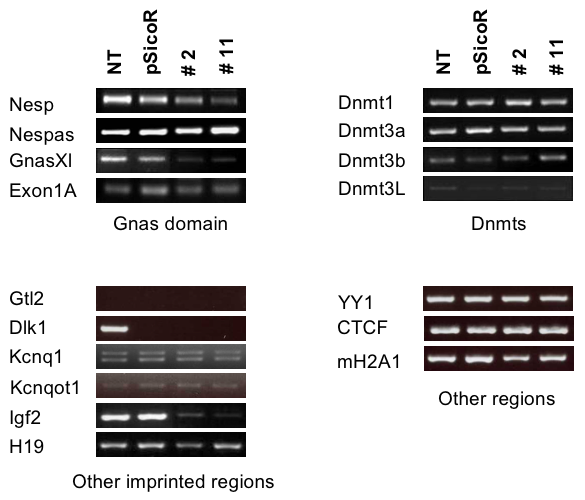

Supplement: Additional file 1 — MacroH2A1 knockdown effects on the transcription of additional genes. The data represent the macroH2A1-knockdown effects on the expression levels of the genes analyzed in this study. [file 1471-2164-8-479-S1.tiff]
